# Current taxonomic status of the cultivable and uncultivable *Paracoccidioides* species

**DOI:** 10.1093/mmy/myae108

**Published:** 2024-11-04

**Authors:** Leonel Mendoza, Raquel Vilela

**Affiliations:** Microbiology,Genetics and Immunology Department, Michigan State University, East Lansing, Michigan 48824, USA; Biomedical Laboratory Diagnostics, Michigan State University, East Lansing, Michigan 48824, USA; Biomedical Laboratory Diagnostics, Michigan State University, East Lansing, Michigan 48824, USA; Faculty of Pharmacy, Federal University of Minas Gerais, Belo Horizonte, Minas Gerais 31270-901, Brazil

**Keywords:** Cultivable and uncultivable *Paracoccidioides* species, taxonomy, nomenclature, lobomycosis

We have read with interest a recent publication that appeared in *Medical Mycology* by Fernandez et al.^1^ The taxonomic status of *Paracoccidioides* species was addressed in that publication. Unfortunately, the publication omitted key references about the latest nomenclatural and taxonomic traits of the genus that deserve to be taken into consideration. In a recent publication Vilela et al.,^2^ introduced the concept of two contrasting populations of *Paracoccidioides* species: cultivable (*Paracoccidioides americana, P. brasiliensis* complex [see below], and *P. lutzii*) and uncultivable *(P. ceti* and *P. lobogeorgii*) species. Although Fernandez et al.,^1^ mentioned the uncultivated species *P. ceti and P. lobogeorgii* referencing Vilela et al.,^3^ the latter name was proposed by Vilela et al., in 2023.^2^ In the 2021 publication Vilela et al.,^3^ introduced the new combination *P. loboi* (Taborda, V.A. Taborda & McGinnis) though the epithet *loboi* was already in use in *Paracoccidioides* (*P. loboi* [Fonseca & Leão] F.P. Almeida & Silva Lacaz) an illegitimate homonym. The illegitimate name, however, was recently corrected with the replacement name *P. lobogeorgii* Vilela, de Hoog, Bagagli & L. Mend.^2^

Surprisingly, Fernandez et al.,^1^ did not mention that *P. americana, P. lutzii, P. restrepoana* [incorrectly cited as *P. restrepiensis*], and *P. venezuelensis* were invalid species.^2,4,5^ The proposed species were invalidated due to nomenclatural mistakes in the original publications that did not follow Arts 40.7: ‘For the name of a new species or infraspecific taxon published on or after 1 January, 1990, of which the type is a specimen or unpublished illustration, the single herbarium, collection, or institution in which the type is conserved must be specified’ and 40.8: ‘For the name of a new species or infraspecific taxon published on or after 1 January, 2019, of which the type is a culture, the protologue must include a statement that the culture is preserved in a metabolically inactive state’ of the International Code of Nomenclature for Algae, Fungi, and Plants (ICNAFP).^2,4,5^ It is important to highlight that besides grouping the genus *Paracoccidioides* into cultivable and uncultivable species, Vilela et al.,^2^ validated the species *P. americana* and *P. lutzii*, a key reference missed by Fernandez et al.^1^ In the same publication they provided also a neotype for *P. brasiliensis* deposited at the *Nucleo de Coleção de Micro-organismos do Instituto Adolfo Lutz, Sao Paulo, Brazil (IAL 9803)*. This was done because Almeida's^6^ original type material of *P. brasiliensis* could not be traced. This neotype provides the genus *Paracoccidioides* with a new tool, not only to support the placement of nearby species, but as a reference isolate for future studies including population genetic, phylogenetic, and genomic analyses.

Previous phylogenetic analysis showed five monophyletic *Paracoccidioides* species.^7^ However, with the inclusion of *P. ceti* in phylogenetic analysis (Bayesian STRUCTURE), Vilela et al.,^2,3^ recognized also five *Paracoccidioides* species, *P. americana, P. brasiliensis*, and *P. lutzii* (cultivable species) and *P. ceti* (affecting dolphins dwelling the Americas Atlantic coastlines) and *P. lobogeorgii* restricted to humans in Latin American countries (uncultivable species). They excluded *P. restrepoana* and *P. venezuelensis* because in Bayesian STRUCTURE and other analyses, these two previously proposed species failed to separate from *P. brasiliensis*, and therefore are now considered part of the former species.^2^ The placement of *P. restrepoana* and *P. venezuelensis* as part of *P. brasiliensis* species explains why Vilela et al.,^3^ did not validate them. More importantly, Turland et al.,^4^ and Borman AM, Johnson^5^ recently called attention to the fact that *P. restrepoana* and *P. venezuelensis* are considered invalid species missing arts 40.7 and 40.8 ICNAFP requirements, alerting the medical mycology community on the status of these species. Surprisingly, Fernandez et al.,^1^ failed to quote these important references.

Regarding the nomenclature of the disease name for the uncultivable species the obsolete name ‘lobomycosis’ should be discontinued.^2^ In a recent publication, Vilela and Mendoza^7^ stated that ‘*To standardize the terminology of fungal diseases, it is recommended that those working with P. lobogeorgii and P. ceti adhere to the current nomenclatural changes to avoid repetition of traditional mistakes*.’ Therefore, the use of paracoccidiodomycosis ceti for the infections caused by *P. ceti* in dolphins and paracoccidioidomycosis lobogeorgi affecting humans is encouraged. Moreover, the term lobomycosis cannot be used for the disease in dolphins since the epithet was introduced for the human disease described by Jorge Lôbo. We understand this is a recommendation to standardize Jorge Lobo's disease name, and therefore it follows an old tradition to unify the disease name from a long list of epithets proposed over the years.^2^

In conclusion, following nomenclatural changes for *Paracoccidioides* species, it is recommended that those working with the cultivable (*P. americana, P. brasiliensis*, and *P. lutzii*) and the uncultivable species (*P. ceti* and *P. lobogeorgii*) to adopt current taxonomic changes, even though in the future these species could be modified, as new tools are proposed to investigate the evolutionary traits of these fascinating group of dimorphic mammalian fungal pathogens.
